# Development of a Laser Powder Bed Fusion Process Tailored for the Additive Manufacturing of High-Quality Components Made of the Commercial Magnesium Alloy WE43

**DOI:** 10.3390/ma14040887

**Published:** 2021-02-13

**Authors:** Stefan Julmi, Arvid Abel, Niklas Gerdes, Christian Hoff, Jörg Hermsdorf, Ludger Overmeyer, Christian Klose, Hans Jürgen Maier

**Affiliations:** 1 Institut für Werkstoffkunde (Materials Science), Leibniz University Hanover, 30823 Garbsen, Germany; klose@iw.uni-hannover.de (C.K.); maier@iw.uni-hannover.de (H.J.M.); 2 Laser Zentrum Hannover e.V., 30419 Hannover, Germany; a.abel@lzh.de (A.A.); n.gerdes@lzh.de (N.G.); c.hoff@lzh.de (C.H.); j.hermsdorf@lzh.de (J.H.); l.overmeyer@lzh.de (L.O.)

**Keywords:** powder bed fusion, magnesium, process development

## Abstract

Additive manufacturing (AM) has become increasingly important over the last decade and the quality of the products generated with AM technology has strongly improved. The most common metals that are processed by AM techniques are steel, titanium (Ti) or aluminum (Al) alloys. However, the proportion of magnesium (Mg) in AM is still negligible, possibly due to the poor processability of Mg in comparison to other metals. Mg parts are usually produced by various casting processes and the experiences in additive manufacturing of Mg are still limited. To address this issue, a parameter screening was conducted in the present study with experiments designed to find the most influential process parameters. In a second step, these parameters were optimized in order to fabricate parts with the highest relative density. This experiment led to processing parameters with which specimens with relative densities above 99.9% could be created. These high-density specimens were then utilized in the fabrication of test pieces with several different geometries, in order to compare the material properties resulting from both the casting process and the powder bed fusion (PBF-LB) process. In this comparison, the compositions of the occurring phases and the alloys’ microstructures as well as the mechanical properties were investigated. Typically, the microstructure of metal parts, produced by PBF-LB, consisted of much finer grains compared to as-cast parts. Consequently, the strength of Mg parts generated by PBF-LB could be further increased.

## 1. Introduction

The role of metals in additive manufacturing (AM) technology has increased in recent years [1]. The production of metal products by AM is divided into three methods, i.e., powder bed systems, powder feed systems and wire feed systems. The advantages of powder and wire feed systems are the possibility to easily repair parts and having higher volume build-up rates. However, the most common method is the powder bed system or powder bed fusion (PBF-LB) according to DIN and ASTM [2]. This method has the ability to produce high-resolution parts with internal passages [3]. The AM technologies do not need any kind of tooling and it is possible to build undercuts. As a result, complex structures can be realized with these methods [4]. New lightweight constructions or individual parts can be realized. Large-volume parts or mass accumulation needed for the casting process can be transformed into framework structures. In this case, the mechanical properties (e.g., stiffness, strength) will be adapted by the framework structure. Ahmadi et al. investigated different structures with regard to their mechanical properties. Their investigations showed that the truncated cube and the truncated cuboctahedron are two of the strongest structures, while the diamond and rhombic dodecahedron structures are comparatively weak. The same behavior was observed for the stiffness. The stronger structures also have a higher stiffness [5]. Additionally, the microstructure strongly depends on the production method. Relating to Trosch et al., as-cast Inconel 718 parts have grain sizes at the scale of 1 mm. With forging, the grain size can be downsized to 10–100 µm. However, with PBF-LB, a grainsize of 1–5 µm can be realized. Additionally, the grain size can be tailored by adjusting the laser parameters. Increasing the scanning speed, for example, leads to a more refined grain size [6]. According to the Hall–Petch relationship, a finer microstructure results in an increased yield strength from 940 (as-cast) to 1185 MPa (PBF-LB) for Inconel 718 [7]. This mechanism translates to other metals and alloys as well and is especially relevant for the strengthening of magnesium alloys.

The PBF-LB process poses several challenges itself. Like the as-cast products, parts manufactured using PBF-LB have residual porosity, and the solution to this issue is dependent on the exact manufacturing process being employed. Kelly et al. optimized the density of Ti6Al4V parts by varying the laser parameters from 96.2% to 99.2%. Thus, the compressive yield strength and compressive yield strain of gyroid structures could be enhanced by 70% and 21%, respectively [8]. Another particularly important challenge with metal powders for AM is the oxide layer present on the powder particles. Due to the high specific surface area of the powder, the proportion of the metal oxide is higher than in other production methods that utilize bulk workpieces. Oxides can prevent the fusion of the particles, especially if they will not melt at the process temperature being used [9]. Significantly higher boiling points of metals in comparison with the melting points of their respective oxide lead to a better processability. The boiling point of metallic titanium (Ti) at 3287 °C is substantially higher than the melting point of the oxide layer (1842 °C). This is similar for iron with a boiling point of the metal of 2861 °C [10]. Mg is problematic in this regard as it has a low melting point of 650 °C and a boiling point of 1093 °C, combined with the oxide’s very high melting point of 2825 °C [10]. This problem can be dealt with by either changing the material composition by alloying or adjusting the laser process itself. In previous investigations, the commercial alloy WE43 emerged as a promising alloy in producing Mg parts with PBF-LB [11]. WE43 consists nominally of magnesium alloyed with 4 wt.% yttrium (Y) and 3 wt.% rare-earth elements (RE). These elements are able to thermodynamically reduce the thermally very stable magnesium oxide (MgO) [10]. In addition, certain rare-earth oxides form an eutectic system with MgO, which has the potential to reduce the melting temperature of the oxide layer by 800 °C [12].

The first investigations to fabricate single tracks out of magnesium using PBF-LB were conducted by Ng et al. (2009) [13]. They proved the feasibility of this process, despite the difference in the evaporation temperature of the oxide compared to the melting temperature of pure magnesium. They also found that the process is highly dependent on the processing parameters, especially the laser power and laser scanning speed. There was an unstable melting process, with much spattering and process emission, diminishing the build quality. In order to improve the process, many investigations were carried out over the next few years to adjust the process parameters for the fabrication of ideal single tracks and completely dense samples [14,15]. Jauer et al. produced the first fully dense sample in 2016. They used a dedicated laser melting system, designed to remove process emissions out of the building chamber. Moreover, they used the magnesium alloy AZ91 to realize samples with a relative density up to 99.5% [16]. However, the problem with aluminum as an alloying component is its poor biocompatibility, which led to increased process development activities for the use of WE43, which has been used in conventionally manufactured resorbable implants previously [15,17].

The objective of the present study was the comparison of the properties of products generated with either PBF-LB or die casting. Hence, the densities, compositions, microstructures and mechanical strengths of the products were investigated.

## 2. Materials and Methods

### 2.1. Magnesium Alloys

**Figure 1:**
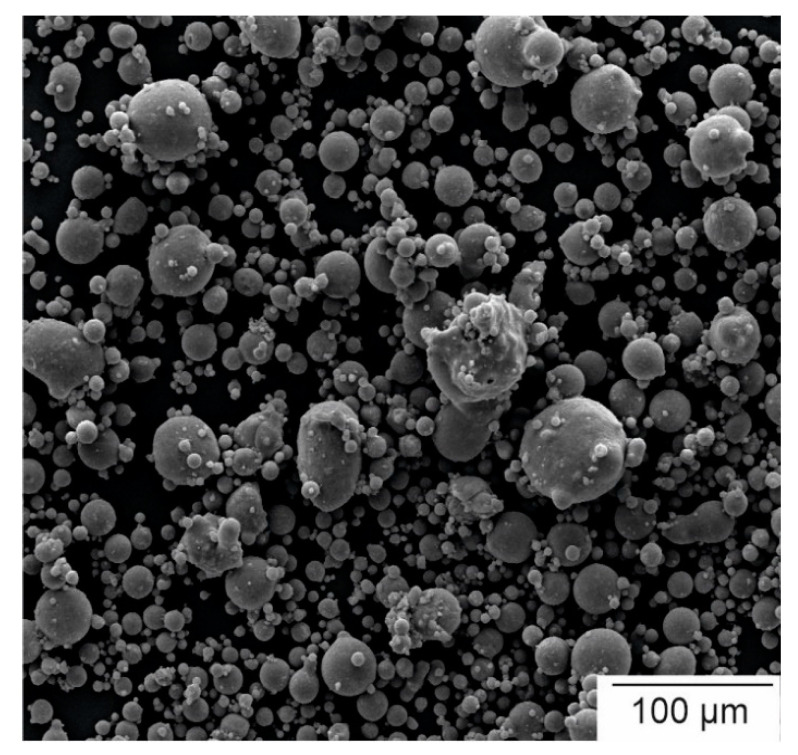
SEM image of the WE43 used for PBF-LB.

In this study, the Mg alloy WE43 (4 wt.% Y and 3 wt.% rare-earth metals, balance Mg) was investigated. For the production of the as-cast samples, WE43 ingots were provided by Magnesium Elektron UK (Manchester). In the PBF-LB experiments, a gas-atomized WE43 powder provided by Carpenter Additives (Widnes, Cheshire, UK) was used. An SEM (scanning electron microscope) image showing a typical selection of powder particles is displayed in Figure 1. The particle diameters are below 63 µm with a median of 9.6 µm and a 0.9 quantile at 18.7 µm. The shapes and the sizes of the powder particles are very inhomogeneous, having, in part, adhering satellites and deformed particles. With the 90% quantile at 18.7 µm, the manufacturing of layer thicknesses down to 20 µm is possible. Moreover, the powder has excellent flowability, which is important even for powder application, and therefore a homogenous melting behavior.

### 2.2. Casting of the WE43 Samples

The reference samples were cast using a gravity die casting process. For the casting process, a mild steel crucible and steel dies (both self-built) were used. The casting geometry inside the dies was a horizontal rod (∅ 20 mm × 220 mm) with a feeder along the top of the rod. Among other things, this geometry was also designed to obtain a homogeneous microstructure. The properties of the samples should consequently be homogeneous along the rod. To prevent interfacial reactions or excessive diffusion of iron into the melt, the crucible was coated with boron nitride (BN). For the die, two different coatings were used. The rod was coated with graphite for a faster cooling rate and the feeder was coated with BN to slow down the cooling rate. The WE43 ingots were then placed into the crucible. For the melting process, a resistance-heated furnace (Nabertherm GmbH, Lilienthal, Lower Saxony, Germany) was used. To shield the Mg melt from oxidation, a constant gas stream of 1000 mL/min of 0.3 vol.% reactive SF_6_ in nitrogen over the top section of the crucible was maintained. The WE43 ingots were melted and the temperature of the melt was then raised to 750 °C. Simultaneously, the dies were preheated to 350 °C. After reaching the casting temperature, the crucible was taken out of the furnace and the melt was cast manually into the preheated dies. Finally, the feeder and sprue were cut off mechanically after demolding. 

The samples for the characterization were prepared out of the as-cast rods (Knuth Wergzeugmaschinen GmbH, Wasbek, Schleswig-Holstein, Germany and EMCO GmbH, Hallein-Taxach, Salzburger Land, Austria). The microstructure of the whole cross-section was analyzed on cylindrical samples (∅ 20 mm × 10 mm) cut out of the rods. The cylindrical samples for the compression tests had a diameter of 5 mm and a length of 7.5 mm. The tensile test specimens had the B4 ×20 sample size according to the standard DIN 50125 and the flat samples for measuring the bending strength were cuboid with dimensions of 43 mm × 6 mm × 2 mm. All these samples were machined out of the rod. 

### 2.3. PBF-LB of WE43

In PBF-LB, a laser melts a multitude of particles in a powder bed, forming layers with a set height. After the melting process, the next layer is applied by first lowering the build platform, applying the powder and melting the layer. This process is repeated until the desired geometry is built. In this process, several parameters have a substantial impact on the resulting geometry and the mechanical properties. A parameter study was executed to establish suitable process parameters for fabrication of the samples. The following parameters were varied: laser power, laser scanning speed, hatch distance, hatch pattern, build plate preheating and layer size.

The samples in PBF-LB were fabricated with a laser melting system of the type SLM125HL (SLM Solutions Group AG, Lübeck, Germany). The specimens were cubes with a size of 5 mm × 5 mm × 5 mm with a 1 mm support structure. The generated samples were analyzed with the statistical software JMP (Version 15, SAS Institute, Cary, NC, USA), in order to gather information in a wide processing interval with the smallest sample possible. A series of three investigations was carried out to determine the processing parameters for the highest relative density with the lowest respective porosity. With the parameters and intervals shown in Table 1, a statistical design of the experiment was developed with the software JMP. The parameter screening was based on a central composite design, which was divided into three parts. The first experiment with 18 sets of parameters was a screening to narrow the possible parameters down to a highly relevant selection. In the following, the best laser parameters were chosen by 36 additional experiments. The best parameters of those experiments were then used for a more detailed analysis in another six additional experiments. 

The objective of this process development was to create an empirical process model of WE43 for the powder bed fusion. This model predicts the relative density from a combination of laser and manufacturing parameters, is calculated with the software JMP and consists of numerous embedded equations. These are based on a selection of parameters in individual equations to show the individual influence and are furthermore crossed to take the interactions between them into account.

### 2.4. Mechanical Testing

The bending strength was determined with three-point bending tests according to the standard DIN EN ISO 7438 using a Zwick Z250kN mechanical testing machine (ZwickRoell GmbH, Ulm, Germany) with a 20 kN load cell. The samples were placed on a two-point bearing surface with a distance of 17 mm. The testing started with the force set to 10 N, followed by the actual bending test with a crosshead speed of 1 mm/min.

The compression tests were performed according to the standard DIN 5016 using a Zwick Z 250 Retro Line mechanical testing machine (ZwickRoell GmbH, Ulm, Germany). The preload used in the compressive test was set to 50 N, followed by a nominal strain rate of 10^−3^ s^−1^.

Furthermore, the behavior of the as-cast samples under tensile load was characterized according to the standard DIN 50125 with the Zwick Z 250 Retro Line mechanical testing machine and a 20 kN load cell. The preload used in the tensile test was set to 25 N.

For each investigated parameter, a minimum number of eight samples were tested to statistically validate the results of the mechanical tests. For the parts generated with AM, the mechanical properties of two separate build jobs were characterized for an indication of the overall reproducibility of the process.

In addition, the Vickers hardness was analyzed according to the standard DIN EN ISO 6507-1 using a Zwick ZHU 250 (ZwickRoell GmbH, Ulm, Germany). The method HV10 was employed using a Vickers indenter and a test force of 98.1 N. On each sample, the average hardness was determined by calculating the mean values of at least seven indentations.

### 2.5. Microstructural Analysis

The as-cast magnesium implants were ground and polished down to a particle size of 1 µm of the polishing agent. Afterwards, the surface underwent a treatment with 10% picric acid in ethanol in order to slightly etch the grain boundaries. The images of the microstructure were taken with a VK-X1000 microscope (Keyence, Neu-Isenburg, Germany). The grain size analysis was carried out with different methods for the as-cast and additive manufactured samples. The microstructure of the parts generated with PBF-LB showed differently shaped grains. To get a meaningful result, the areas of the individual grains were determined. Therefore, the grain boundaries were marked separately to calculate the inner areas. The as-cast samples, however, had mostly globular grains and hence the grain sizes of the as-cast samples were analyzed according to the ASTM E 112 standard. Using the calculated grain size, the occupied area of the grains was calculated with the assumption that the grains were spherical. The scanning electron microscope (SEM) images of the microstructure and the energy-dispersive X-ray spectroscopy (EDX) analysis were obtained with a SUPRA 55 VP (Carl Zeiss AG, Oberkochen, Germany) using an acceleration voltage of 12 kV, a spot size of 2.5 nA, a working distance of 6.2 mm and magnifications of 50×, 250× and 400× (as-cast) and 50×, 500× and 6000× (PBF-LB).

### 2.6. X-ray Characterization

Phase determination was carried out by X-ray diffraction (XRD) using a C8 Discover (Bruker Corporation, Billerica, MA, USA) with CuKα radiation (λ = 1.540562 Å). The samples were scanned continuously from 15° to 88° in 2θ with a step size of 0.01°.

The porosity of the as-cast samples compared with the AM-generated parts with different process parameters was investigated with an Xradia 520 Versa X-ray microscope (Carl Zeiss AG, Oberkochen, Germany). The images were taken with a CCD camera (Andor Technology Ltd., Belfast, Northern Ireland, UK) and lenses at 4 times magnification. For the as-cast samples, a spatial resolution of 2.4 µm, an acceleration voltage of 70 kV, a power of 6 W and an exposure time of 3.2 s were used. The tomographic images of the parts generated with AM were implemented with spatial resolutions of 5 (samples for the compression test) and 6 µm (samples for bending tests), an acceleration voltage of 80 kV, a power of 7 W and exposure times of 5 (samples for the compression test) and 8 s (samples for bending tests).

## 3. Results

### 3.1. Process Development

**Figure 2:**
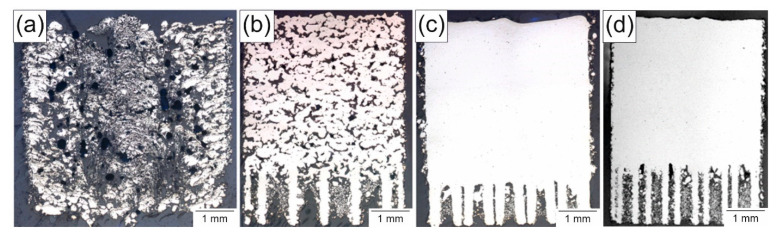
Cross-sections of representative specimens from the process screening (**a**–**c**) with the parameters shown in Table 2; best overall result of this process development based on the final empirical process model (**d**).

The objective of the process development was to create a process model to be able to determine a set of parameters adjusted to the fabrication of high-quality WE43 parts. This process model links the laser and manufacturing parameters via empirical equations to the relative density as the target variable. Figure 2a–c show three specimens in a cross-section view, displaying the influence of the energy input by the laser on the relative density. These cross-sections as a base for analysis are oriented perpendicular to the manufactured layers to obtain detailed information about the course of the building process. This allows faults of the melting behavior to be detected in individual layers and to, furthermore, be correlated with laser exposure strategies. The corresponding process parameters are given in Table 2. In addition, the set of parameters given by the model for a maximum density, which was also used to process the samples in the present study, was added in Figure 2d and as #d in Table 2. It is shown that the forecast of the process model is valid and the calculated parameter set for maximum density leads to 99.9% density.

The specimen shown in Figure 2, option a, clearly had an energy input which was too high. If the energy input is too low, dense samples could not be realized either, as is seen in Figure 2, option b. A correct energy input leads to dense samples with a porosity of less than 0.1%. Figure 2, option c, corresponds to the best result of the parameter screening and had 99.9% relative density. This specimen had sufficient density, but showed poor dimensional accuracy. The layer height of 75 µm is desirable due to the good build-up rate but results in a significant stair chase effect on angled surfaces. Thus, a parameter with a high density for a smaller layer height had to be identified, and the statistics software was used to create an empirical process model. To simplify the model and the calculation, some processing parameters were fixed to eliminate variables. The parameters were set to 20 µm layer height, 40 °C build plate preheating and chess hatching with a field size of 4 mm edge length as a laser exposure with a 90° hatch rotation from layer to layer. With these parameters as a basis, the next experiment investigated the influence of the laser power, scanning speed and hatch distance in detail. The selection was made due to experience in preliminary work, where these three variables had the most significant impact. Processes could be optimized to very good results, disregarding the layer height, preheating temperature and laser exposure strategies due to their minor influence. The laser power was varied between 20 and 100 W, the scanning speed from 100 to 1500 mm/s and the hatch distance from 10 to 150 µm. The parameters used were based on the forecast of the process model.

In this parameter interval were six parameter combinations, which led to relative densities of 99.9%. The best result is #d in Table 2, which showed a very high density and a good dimensional fit. This parameter set was then used for the fabrication of the samples for the mechanical testing as well as the microstructure investigation.

### 3.2. Tomographic Analysis

**Figure 3:**
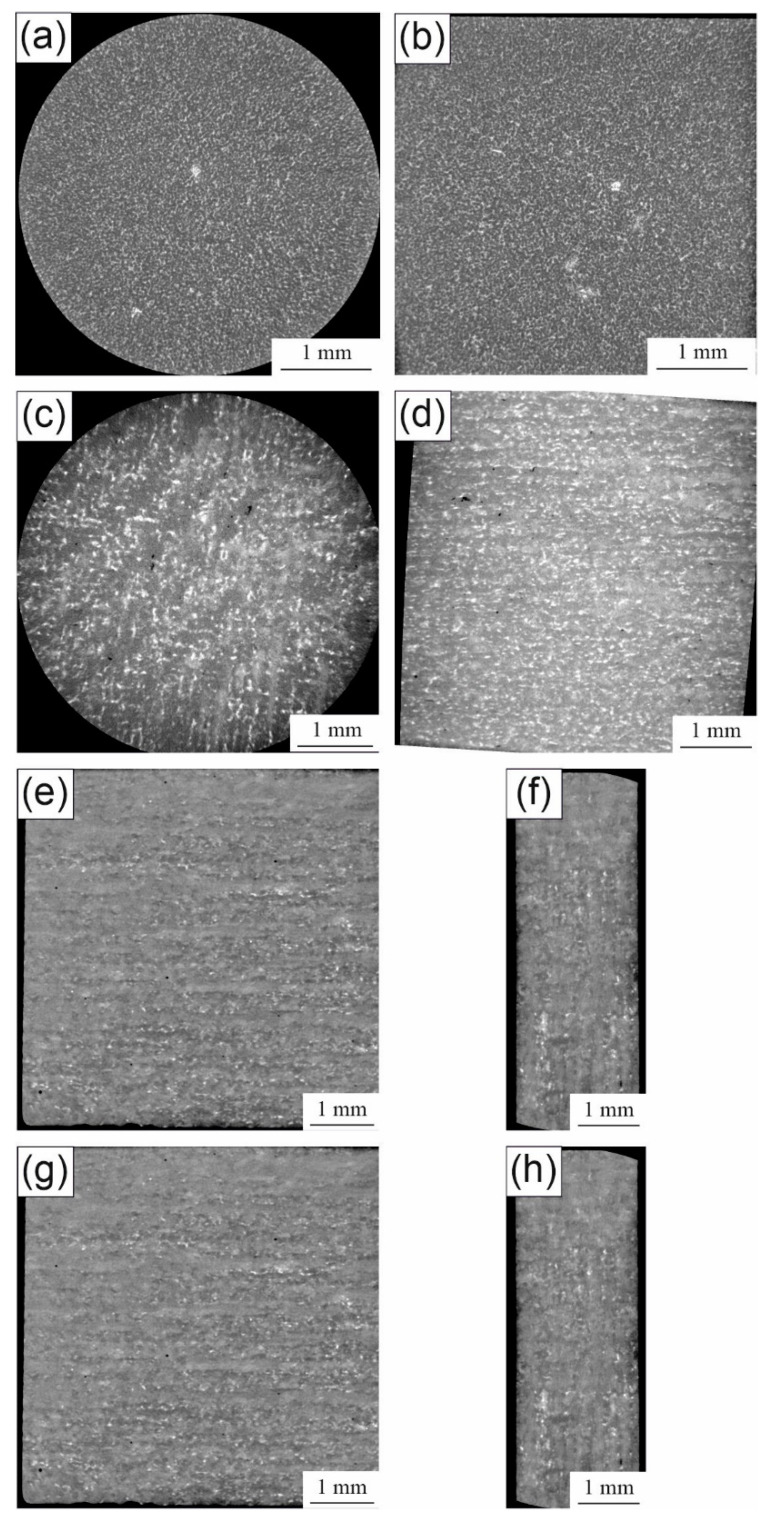
2D images from the tomographic measurements of (**a**,**b**) as-cast WE43, (**c**,**d**) PBF-LB-generated WE43 samples for the compression test, (**e**,**f**) first PBF-LB-generated sample for the bending tests and (**g**,**h**) second PBF-LB-generated sample for the bending tests.

The spatial distributions of the rare-earth precipitates and the pores were determined by volumetric XRM scans. Typical cross-sections from two different planes of cast and additively manufactured parts are shown in the 2D slice images in Figure 3. In the as-cast samples (Figure 3a,b), no pores were detected, which means the pores are smaller than the detection limit or no pores exist in the investigated area. In addition, the rare-earth-containing phases in the microstructure, which are the brighter spots in the tomographic images due to higher X-ray attenuation, are in the micrometer range with only a few agglomerated spots of those rare-earth precipitates. Clearly, it was demonstrated that the precipitates have no preferential orientation. In comparison, pores were detected in every sample of the parts generated with PBF-LB (Figure 3c–h). The cylinders for the compression tests had smaller spherical or elongated pores, which were arranged between the layers. The size was in the range of 20 to 100 µm, whereas elongated pores reached a length of up to 160 µm. Considering the precipitates, those were also larger than in the as-cast samples and were arranged in layers. Rare-earth precipitates emerged preferably at grain boundaries [18]. Thus, smaller and uniformly distributed precipitates may have resulted from smaller grains. Considering the cuboid-shaped PBF-LB samples for the bending test, the porosity varied strongly with the building process, although the same parameters were used. In the first process, the porosity was low and similar to the smaller samples for the compression test. By producing the same cuboid-shaped samples in a second process, the porosity increased strongly. However, only the amount of pores increased; their size remained the same. In addition, the layered structure of the process was visible based on the distribution of the precipitates. The porosity strongly affected the mechanical behavior as will be demonstrated later.

### 3.3. Microstrucure and Phase Analysis

**Figure 4:**
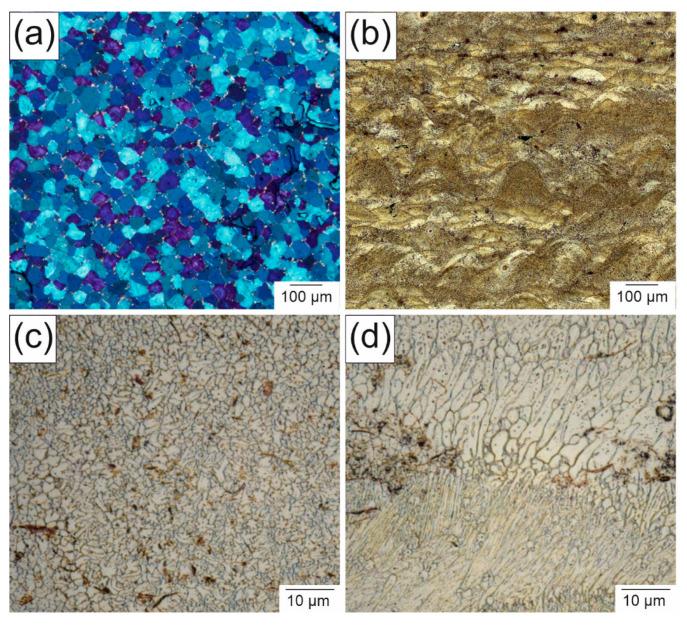
Light microscopy images of the microstructure of WE43: (**a**) as-cast, (**b**) PBF-LB-generated and (**c**,**d**) detailed images of different spots in (**b**).

The microstructure of the WE43 specimens strongly depended on the manufacturing process, as is shown in Figure 4. In the as-cast state (Figure 4a), the grains were comparatively large with mostly globular shapes and a mean grain size of 22 ± 0.5 µm. To be able to compare the grain size of the as-cast state with the PBF-LB state, the area was calculated on the assumption that the grains are spherical. In this case, the grains had an area of 380 µm^2^. Unlike the porosity, the microstructure of all parts generated with PBF-LB was similar, which is why only representative images of the individual areas are shown in the following. The microstructure of the PBF-LB parts must be considered at two levels. At a mesoscopic level, the structure consists of parabolic areas with different sizes (Figure 4b). The parabolic structure was due to the melting of small separate spots and shows the direction of solidification. Each melt pool solidified in the same direction that the laser scanned along the surface. If pores occurred, they appeared mostly at the borders of the parabolic structures. This also indicates that the scanning parameters can influence the porosity strongly. In contrast to the as-cast condition, the grains of the PBF-LB parts consisted of much smaller and arbitrarily shaped grains. In the middle of the parabolic structures, the grains were more spherical (Figure 4c), while the grains on the borders of the parabolas could be both spherical or elongated (Figure 4d), whereby the latter grains were more pronounced and were visibly larger. Due to the elongation of the grains, a meaningful average grain size could not be determined using the same procedure as for the as-cast samples.

**Figure 5:**
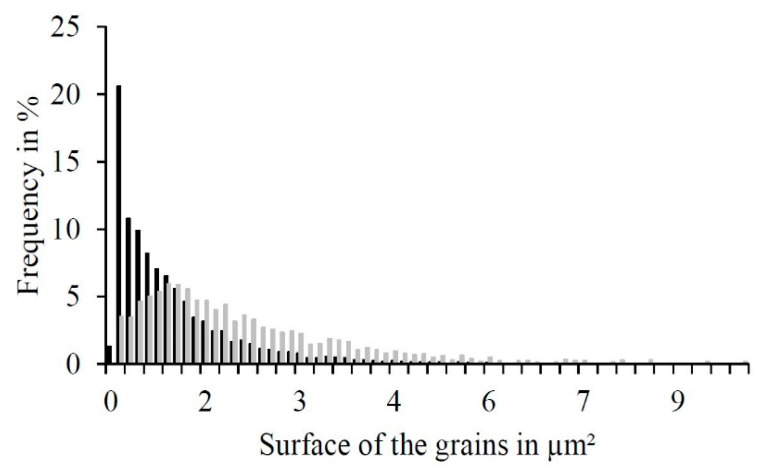
Histogram of the area-based grain size of PBF-LB-generated WE43 of the spherical grains (**gray**) and longitudinal grains (**black**).

Thus, the surface of the grains was analyzed and plotted in a histogram (Figure 5). Concluding from the numeric proportion, it is apparent that a majority of the grains were spherical ones in the micron or submicron range, as shown in Figure 4c. Towards the larger grains, the numeric histogram flattened out slowly due to the elongated grains displayed in Figure 4d. Although the amount of smaller grains is significantly higher, the area proportion of the elongated grains was similar to that of the spherical grains, as represented by the surface area histogram. Hence, the two grain geometries in Figure 4c occurred with an equal share of the surface area.

**Figure 6:**
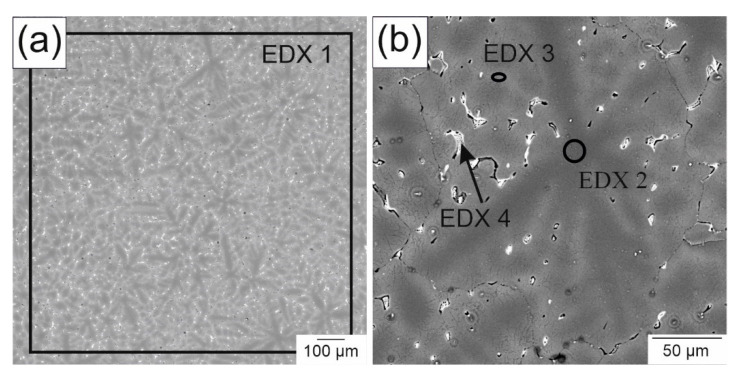
Backscattered electron images of as-cast WE 43 with (**a**) low and (**b**) high magnification.

**Figure 7:**
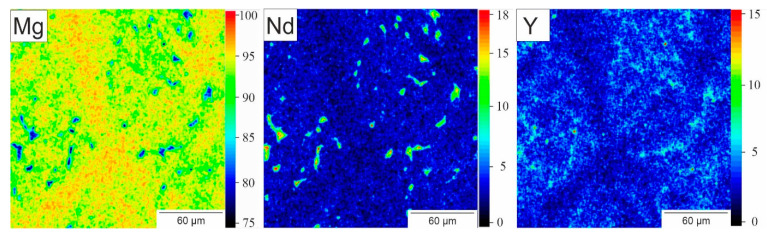
Element mapping of Mg, Nd and Y of the specimen in section in 6b.

The different phases and their composition were characterized in SEM images along with additional EDX measurements. The backscattered electron images of the as-cast WE43 with the additional spots of the EDX measurements are shown in Figure 6 with a 50× (Figure 6a) and 250× (Figure 6b) magnification, together with the compositions of the different areas in Table 3. As the backscattered electrons show an element contrast, the distribution of the rare earths is qualitatively visualized. The overview image shows a needle-like structure of the Mg matrix, separating areas with a higher amount of rare-earth metals. In addition, precipitates, visible as small bright spots, were uniformly distributed. Both the precipitates and the separated parts in the matrix were homogeneously distributed. With the EDX measurement, only neodymium (Nd) could be detected out of the elements of the rare-earth mixture and had the expected magnitude of 2.4 wt.%. In contrast, the yttrium (Y) content was almost double the target value, with 7.9 wt.%. However, the tested volume was comparatively small, which is why an additional spark spectrometer measurement was employed. In this way, a larger volume of the sample was measured and the data were close to the nominal concentrations. Looking at the detailed image (Figure 6b), the center of the needle-like structure was in the middle of the grains. It was also found that most of the Nd was in the precipitates (EDX 4), whereas yttrium was concentrated in the separated part of the matrix. These results were confirmed for the overall structure through EDX mappings (Figure 7) of the section in Figure 6b. The Nd content in all precipitates was found to be the same as was measured on a single precipitate (EDX 4). In contrast, Y did not show a significant increase in the precipitates compared to the Y found in the matrix. Due to the higher resolution of the SEM compared to the XRM, the pores, which are represented by the black dots in Figure 6a, could be revealed.

**Figure 8:**
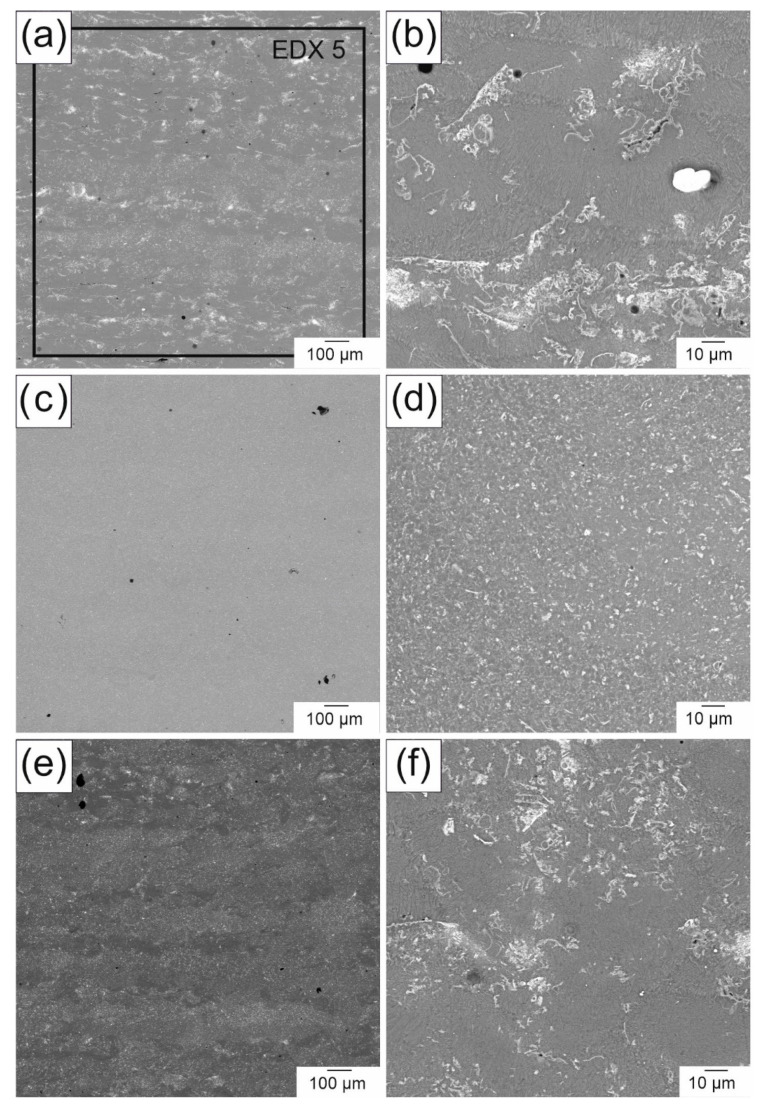
Backscattered electron images of PBF-LB-generated WE43: (**a**,**b**) sample for the porous bending test and (**c**–**f**) sample for the compression test.

**Figure 9:**
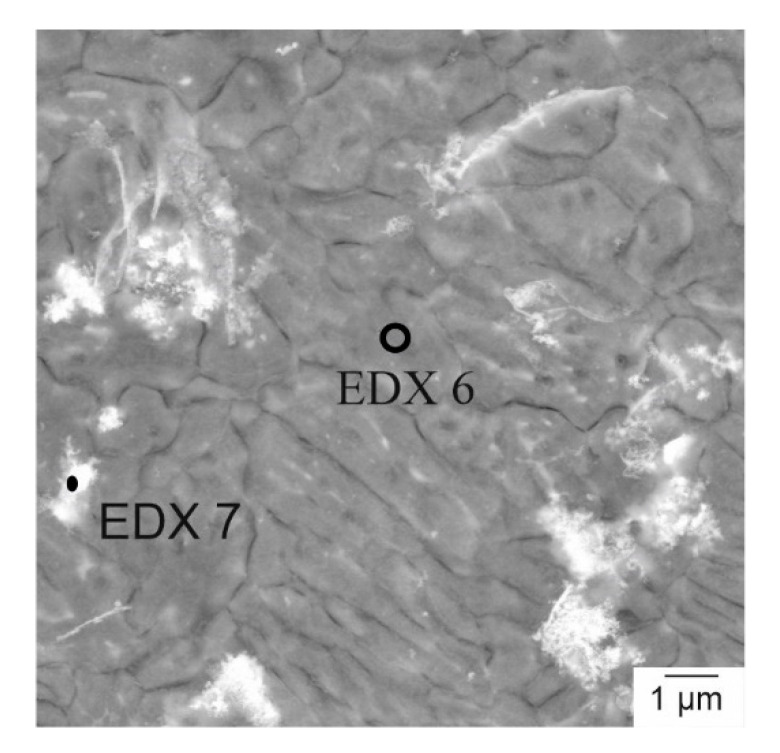
Backscattered electron images of PBF-LB-generated WE43 with the areas of the EDX measurements.

Unlike the grain structures of the parts generated with PBF-LB, the phases of these parts were arranged differently. In the samples for the bending tests (Figure 8a,b), most of the precipitates were inhomogeneously distributed. Only a few layers consisted of evenly distributed precipitates. The higher-magnification image (Figure 8b) shows that the precipitates were agglomerated in some areas, but they still formed mostly fine structures in those agglomerations. Only in some cases did clusters of precipitates occur. In contrast, large parts of the compression test samples (Figure 8c,d) contained small and homogeneously distributed precipitates. However, there were still areas with agglomerated precipitates similar to the cuboid samples, as shown in Figure 8e,f. Although, locally, a homogeneous structure could be realized, the process was still not stable enough to produce a large volume with a homogeneous structure and a density above 99%. As with the as-cast samples, the composition of the different phases was also analyzed in the PBF-LB-generated parts. Besides the spots in Figure 9, a large-area EDX measurement (EDX 5) of Figure 8a was conducted. The compositions of the different phases, independent of the samples, were similar to each other, which is why only a representative EDX analysis of one PBF-LB-generated part is shown in Table 4. The Nd and Y content is rather high in the large-area EDX measurement. To determine the impact of the process on the composition, an additional EDX measurement of the initial powder was conducted on several particles. The RE content in the initial powder was actually found to be lower than in the final produced parts. As was mentioned before, the problem with Mg in the PBF-LB process was the low boiling point of metallic Mg. During the heating of each spot, Mg could have been vaporized to a greater extent than during the casting process.

In the matrix, there was also a variation in the RE content similar to the as-cast sample. Due to the finer microstructure, the separate parts were thinner. Hence, an accurate composition was hard to obtain because the excitation volume could easily include RE-rich areas. Consequently, the measured RE content of the matrix is much higher than the solubility limit. In contrast to the as-cast sample, the Y content in the precipitates was remarkably raised instead of the Nd content. According to the phase diagrams, Nd-rich precipitates should occur. However, the cooling rate in the PBF-LB process was much faster. The resulting intermetallic phase of Nd and Mg has a lower diffusion rate and could not be separated into a second phase. Thus, the formation of Nd-rich precipitates is kinetically inhibited.

**Figure 10:**
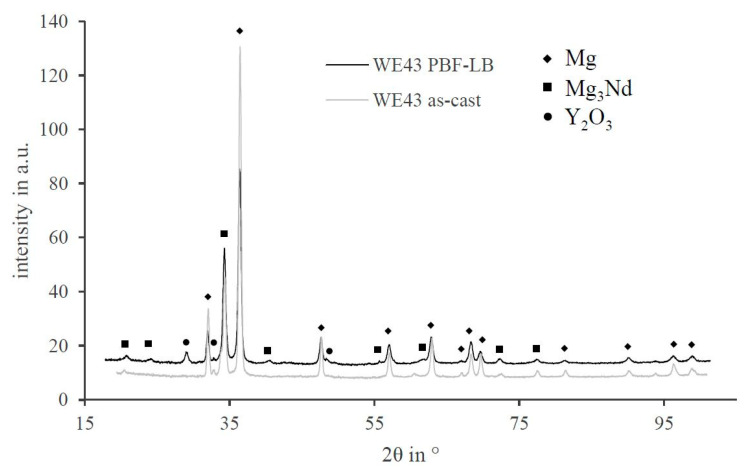
XRD pattern of as-cast and PBF-LB-generated WE43 with the corresponding phases.

The XRD analysis shows the occurring phases in the PBF-LB and as-cast samples, c.f. Figure 10. As expected, the Mg peaks were the most distinctive ones. Besides Mg, the phase Mg_3_Nd occurred in samples of both production methods, although those precipitates were not found in the SEM/EDX analysis of the PBF-LB-generated parts. Therefore, the formation of Mg_3_Nd was not kinetically inhibited, but rather had a diffusion-based formation of the precipitates. Furthermore, also striking is that only the oxide of yttrium (Y_2_O_3_) could be detected, especially in the PBF-LB parts, which is most prominent in the peak at 29°. Those distinctive peaks could not be detected in the as-cast samples. Consequently, the formation of oxides was process-based and did not emerge after the preparation of the samples. The Y-rich precipitates in the PBF-LB samples could consequently be Y_2_O_3_. Unfortunately, the oxygen content could not be quantified with the EDX measurement before, which is why the precipitates of Y_2_O_3_ could not be localized by the SEM-EDX analysis. However, only Y_2_O_3_ was found as a Y-rich phase in the PBF-LB-generated parts. Consequently, those precipitations in Figure 9 should be Y_2_O_3_. This originates from the comparatively high amount of oxides in the powder-based process, as it was mentioned before.

### 3.4. Mechanical Properties

**Figure 11:**
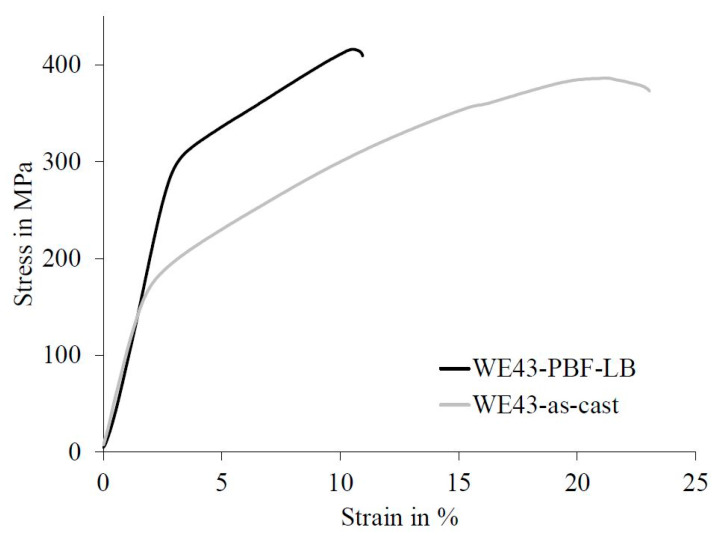
Stress–strain diagram of as-cast and PBF-LB-generated WE43 under compressive load.

**Figure 12:**
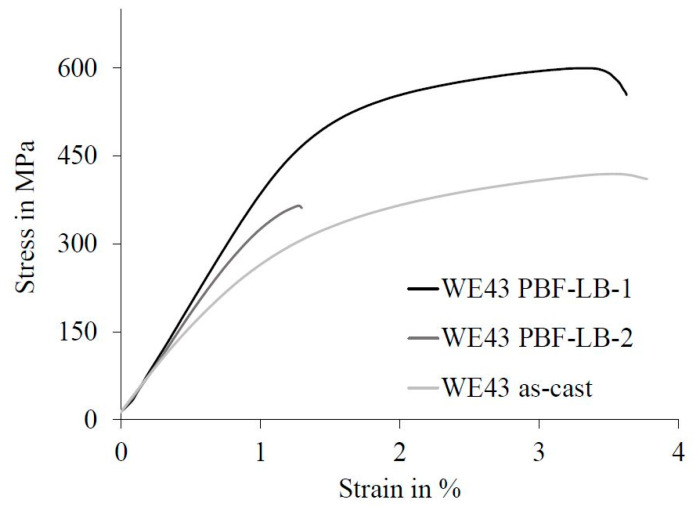
Stress–strain diagram of as-cast and PBF-LB-generated WE43 under bending load.

The mechanical properties are a key factor for lightweight constructions, especially for AM-generated parts, as they can be designed without considering the manufacturability by casting or forming. On this account, the as-cast and PBF-LB-generated parts were tested under compressive and bending stresses. However, up until now, the behavior under tensile stress was only tested with the as-cast samples. As it was mentioned before, the building direction of the samples for the mechanical testing generated by PBF-LB was along the weakest direction. The stress–strain diagrams of the behavior under compressive and bending loads are shown in Figure 11 and Figure 12, respectively. The corresponding characteristic values of the compressive, bending and tensile tests and the hardness are listed in Table 5. The elastic behavior of WE43 does not depend on the production method, i.e., parts produced by PBF-LB and gravity die casting had similar elastic moduli. However, the elastic modulus is a physical quantity and depends on the bonding strength of the atoms, whereas the strength strongly depends on the microstructure, which was influenced by the production method. The as-cast samples had a comparatively low yield strength, which leads to an early onset of plastic deformation. Plastic deformation of PBF-LB-generated WE43 parts started at double the applied stress when compared to the as-cast parts. The compressive strength of PBF-LB-produced parts was also higher, but the difference was less pronounced. However, the ductility of the as-cast parts was higher as compared to PBF-LB, as shown in Figure 11. In fact, the elongation to fracture was doubled when using the as-cast parts. The samples showed little variation in mechanical behavior, so only one representative stress–strain diagram is represented for each case in Figure 11.

The elastic deformation under bending load was similar in both build jobs of the PBF-LB-generated parts. However, the plastic deformation behavior differed significantly. While the first samples generated had a high yield strength and, additionally, a high bending strength, the samples generated in the second manufacturing process clearly had a low proportion of plastic deformation, which also begins at a much lower stress. The preparation of the samples for the bending test was not yet reproducible. The bending behavior ranged from brittle to ductile. In particular, the ductility of the PBF-LB parts with a low porosity (WE43 PBF‑LB‑1) was more similar to the as‑cast samples under bending load than under compressive load. However, the bending strength of the as-cast WE43 was considerably lower.

Magnesium typically shows a tension pressure anomaly [19], which was partly observed in the present study. The yield strength was similar in the as-cast WE43. Only the compressive strength strongly increased compared to the tensile yield strength by 108%. The biggest difference, however, was the elongation to fracture. It is expected that the strength of WE43 generated with PBF-LB under tensile stress should increase similarly to the compressive strength, but this is still to be investigated.

Strengthening of a material by a finer-grained microstructure typically results in an increase in hardness. The same behavior could be observed in the present study. As shown in Table 5, the WE43 parts generated by the PBF-LB process had an increase in hardness by 36% as compared to the as-cast condition.

## 4. Discussion

In the present study, WE43 parts of high quality could be successfully generated by employing PBF-LB. By further adjustments to the process, a density of over 99.9% could be achieved. The laser power, the scanning speed and the hatch distance had a big influence on the quality of the produced parts. So far, densities of at least 99% have been achieved when using WE43 for PBF-LB by generating small cubes with an edge length of 3 mm. If the dimensions of the samples were increased (edge length of 5 mm) or a more complicated geometry had been used, the density was observed to decrease to 95% according to Gieseke [11]. The process development resulted in a parameter set, which increased the density up to 99.9% for parts in the size range between 5 and 10 mm. However, when the dimensions were increased significantly to 50 mm, the porosity could not be set reproducibly. The reason is suspected to be a change in heat conductivity and can be mitigated by further investigation of processing parameters in the desired volume. Therefore, it is recommended to tailor the specimen in testing to a similar geometry for the desired applications. Since in a process development of a novel material, the feasibility has to be tested first, this work is the first step in this direction. As a consequence, the abstractability must be improved by a better understanding of the melting and solidification behavior. Using the same set of parameters in different building jobs leads to different porosities. In large volume parts, the large amount of pores should be homogeneously distributed because the set of parameters was equal throughout the whole sample [8]. The tomographic images of the PBF-LB parts show a deviating behavior. The pores are not homogeneously distributed in the whole volume, which suggests that the process is not stable throughout. Possible solutions are widening the process window by developing a new process variant or by tailoring the Mg alloy towards the process’s requirements. Even in a stable process with a minimized porosity, the number of pores is still higher compared to the as-cast samples. However, the PBF-LB samples of different sizes showed the same porosity when the process was stable. This suggests that the porosity is independent of the size. In contrast, the porosity of as-cast samples strongly depends on the thickness of each part. Thicker parts show higher shrinkages, followed by an increase in the pore size and porosity [20].

The phase analysis had revealed that in both cases, the material consisted mostly of Mg with Mg_3_Nd precipitates. This is contrary to the expectations based on the phase diagram because the thermodynamically stable phase for low amounts of Nd in Mg is MgNd [21]. Dealing with the oxide layer is a key issue for Mg alloys in the PBF-LB process. Keeping this in mind, the only oxide phase that emerged with a detectable level was Y_2_O_3_. In the case of a mixture of MgO and Y_2_O_3_, the melting point of the oxide layer should be between 2046 (eutectic temperature) and 2401 °C (melting point of Y_2_O_3_) [12]. Comparing the two XRD patterns, the issue of the oxide content was clearly demonstrated. The higher yttrium fraction in the precipitates of the PBF-LB parts was most likely caused by the oxide because no other yttrium-containing phases were detected. Comparing all the oxides of the alloying elements, Y_2_O_3_ is thermodynamically the most stable one. In fact, yttrium is able to reduce the oxides of the other alloying elements [10].

The grain structure of the PBF-LB parts is independent of the size or the quality of the build job. The parabolic structure, which has its origin in the beam traces, is strongly pronounced. This typical process-related structure could also be found in the work of Wei et al., who produced AZ91 parts by AM [22]. Whether and to what extent the beam traces and the layered structure appear strongly depend on the material and on the process parameters. Thus, in CoCrMo alloys, the parabolic structure is very prominent. In contrast, Ti alloys, for example, can prevent the formation of this layered structure due to grain growth across the layers [23], although it is possible to reach a trace structure with Ti6Al4V by adjusting the PBF-LB process with the disadvantage of a high porosity [24]. Comparing the grain size of the AM-generated parts with the as-cast parts, the difference in grain size is of two orders of magnitude, which is typical when comparing both processes. The same behavior was investigated by Trosch et al. by comparing the grain size of AM parts, formed parts and as-cast parts. In their study, the grain size was three magnitudes higher than the AM parts. Even the forged parts had 10 times larger grains [7]. Zumdick et al. produced WE43 parts through AM that had spherical-like grains with a diameter of 1.1 µm. They also described the parabolic structure, but not with the elongated grains found in the present study [25]. In as-cast samples, such elongated grains are typical with an anisotropic solidification from the mold wall to the center [26]. Similarly, the solidification in the PBF-LB parts is vertical to the parabolic line, as if it were one separated melt pool that solidifies inwards. Those separated melt pools also have spherical grains in the center emerging in a homogeneous nucleation, shown in the work of Dahle et al. [26]. In the present study, the volume share of both parts was roughly equal. Using the influence of the process parameters, the properties can be adjusted to the component’s requirements.

This principle was used by Eifler in his work to adjust the mechanical properties and the corrosion behavior of the Mg alloy ZNdK100 by adjusting the microstructure with different extrusion parameters. The resulting microstructure varied from a completely recrystallized structure with only small grains to a partly recrystallized structure with a bimodal grain size distribution of deformed grains of the as-cast state and recrystallized grains. The strength, for example, could be raised to over 350 MPa [27]. The mechanical properties are particularly important for lightweight constructions. Seitz et al. investigated different extruded Mg alloys with the alloy LAE442, showing a maximum in the bending strength of 570 MPa and a compressive yield point of 150 MPa [28]. The as-cast WE43 samples had a similar compressive yield strength but a significantly lower bending strength. The strength of WE43 can also be increased by extrusion, as was performed by Dieringa et al. They reached a compressive yield strength of 261.5 MPa and a compressive strength of 420.4 MPa. However, the PBF-LB parts had even higher strengths than those from extruded LAE442 and WE43. This could be attributed to the even smaller grain size of the PBF-LB process compared to casting and extrusion, which increases the strength due to the Hall–Petch effect [7,29]. Using the process to vary the microstructure of PBF-LB parts as Thijs et al. did for Ti6Al4V, the properties of additive manufactured Mg could also be adapted to the application [24].

The problems with Mg stem from its difficult processability, which results from the thermophysical properties of Mg and MgO; therefore, the useful process window is strongly limited. By developing a new process and a new alloy tailored to the special properties of Mg, the process window could be widened, which could stabilize the process and increase the parts’ properties.

## 5. Conclusions

The present study demonstrated that dense magnesium parts with relatively high volumes can be produced by PBF-LB with the alloy WE43. The main results can be summarized as follows:

1. With a process development targeted for maximum relative density, the process can be stabilized to generate large parts while ensuring a high density.
2. The microstructure consists of a bimodal grain size distribution with smaller spherical grains and larger elongated grains. Using the laser parameters, the microstructure could be adapted to directly adjust the properties of the generated part. Compared to the as-cast state, the grain size is one to two orders of magnitude lower, which explains the high strength even for WE43.
3. Still the porosity of the PBF-LB parts is higher than in the as-cast parts, which reduces the strength. Thus, there is still a great potential in the PBF-LB process. In further investigations, additional adjustments, such as those to the laser parameter or the atmosphere, have to be made to stabilize the process for Mg. Furthermore, an alloy adapted to the process could enhance the process capabilities.
4. The PBF-LB parts mainly consist of Mg, Mg_3_Nd and Y_2_O_3_. The objective of reducing MgO to Mg by the rare-earth elements could be achieved. Due to the fast cooling rate, no Mg_3_Nd precipitates could be formed. With additional heat treatments, precipitates of these intermetallic phases could be realized and additionally change the components’ properties.

## Figures and Tables

**Table 1:** Parameters for the central composite design of the first parameter screening.

Step	Process Parameters for Screening Experiment					
	Laser Power in W	Scanning Speed in mm/s	Hatch Distance in µm	Layer Height in µm	Hatch Pattern	Build Plate Temperature in °C
−1	20	100	10	20	Lines	40
0	60	800	80	-	-	-
1	100	1500	150	75	Chess	200

**Table 2:** Set of parameters used for processing of the specimens shown in Figure 2.

Option	Process Parameters							
	Laser Power in W	Scanning Speed in mm/s	Hatch Distance in µm	Layer Height in µm	Hatch Pattern	Build Plate Temperature in °C	Energy Input in J/mm^3^	Relative Density in %
a	20	100	10	20	Lines	200	1000	42.2
b	60	800	80	75	Lines	200	12.5	80.0
c	100	800	10	75	Chess	200	625	99.9
d	80	450	45	20	Chess	40	197.5	99.9

**Table 3:** Results of the EDX measurements indicated in Figure 6.

Element	Composition in wt.%				
	EDX 1	EDX 2	EDX 3	EDX 4	Spark Spectrometer
Mg	89.7	97.8	94.9	84.0	94
Y	7.9	1.0	3.0	4.7	3.9
Nd	2.4	1.2	2.1	11.3	2.1

**Table 4:** Composition of the EDX measurement of the spots in Figure 8 and Figure 9.

Element	Composition in wt.%			
	EDX 5	EDX 6	EDX 7	WE43 Powder
Mg	89.4	91.5	73.0	91.6
Y	6.2	4.4	22.1	4.6
Nd	4.4	4.1	4.9	3.8

**Table 5:** Mechanical properties of as-cast and PBF-LB-generated WE43 parts.

Load Type	Characteristic Value	WE43 as-Cast	WE43 PBF-LB	
Compressive load	Yield strength in MPa	146 ± 7	297 ± 8	
	Compressive strength in MPa	383 ± 37	424 ± 41	
	Elongation in %	20 ± 2	11 ± 2	
Bending load	Yield strength in MPa	271 ± 35	499 ± 10	359 ± 18
	Bending strength in MPa	430 ± 30	601 ± 31	375 ± 24
Tensile load	Yield strength in MPa	142 ± 2	-	-
	Tensile strength in MPa	184 ± 22	-	-
	Elongation in %	2.3 ± 2	-	-
Hardness	Vickers hardness in HV10	69.0	94.1	

## Data Availability

The data presented in this study are available on request from the corresponding author. The data are not publicly available due to privacy.
